# Lead Accumulation in the Straw Mushroom, *Volvariella volvacea,* from Lead Contaminated Rice Straw and Stubble

**DOI:** 10.1007/s00128-013-1025-4

**Published:** 2013-06-09

**Authors:** Thapakorn Kumhomkul, Thanawan Panich-pat

**Affiliations:** Environmental Science and Technology Program, Department of Science, Faculty of Liberal Arts and Science, Kasetsart University, Kamphaeng Saen Campus, Nakhon Pathom, 73140 Thailand

**Keywords:** Lead, Straw mushroom, Rice substrate, *Volvariella volvacea*, Food contamination

## Abstract

Straw mushrooms were grown on lead contaminated rice straw and stubble. Study materials were dried, acid digested, and analyzed for lead using flame atomic absorption spectrophotometry. The results showed the highest lead concentration in substrate was 445.350 mg kg^−1^ in Treatment 3 (T3) and the lowest was BD (below detection) in Treatment 1 (T1). The maximum lead content in straw mushrooms was 5.072 mg kg^−1^ dw in pileus of T3 and the minimum lead content in straw mushrooms was BD in egg and mature (stalk and pileus) stage of T1. The lead concentration in straw mushrooms was affected by the age of the mycelium and the morphology of mushrooms. Mushrooms’ lead uptake produced the highest accumulation in the cell wall. Some lead concentrations in straw mushrooms exceeded the EU standard (>3 mg kg^−1^ dw).

Straw mushrooms (*Volvariella volvacea*) have been a popular delicacy in many countries, and the estimated production in 2003 was approximately 6,000,000 tons in China, accounting for about 5 % of the total world production (Buswell and Chen 2005). Mushrooms have high nutrient content such as protein, vitamins, fats, amino acids and other constituents with medicinal values. However, some mushrooms were reported with lead (Pb) accumulation, due to metal uptake from contaminated substrate by the spacious mycelium of straw mushrooms (Kalač 2009; Nilanjana 2005). Lead is an element that has the potential to cause devastating health effects such as neurological damage, blood disorders, hypertension and renal impairment (Jitrapun et al. 2007; Sroisiri et al. 2005).

In Thailand, the problem of Pb contaminated soil in the paddy fields of Klity village, Kanchanaburi province was reported in 1998. The cause of the contamination was untreated wastewater from mine ore-dressing, discharged into Klity Creek and distributed into soil (Sroisiri et al. 2005). Rice cultivated in lead contaminated paddy field absorbed and accumulated lead in straw and stubble, which can be used for straw mushroom cultivation. The purpose of this study was to measure lead accumulation in the pileus and stalk of straw mushrooms grown in lead contaminated rice straw and stubble.

## Materials and Methods

The experimental protocol followed a completely randomized design (CRD) with five replications per treatment. Baskets used to cultivate straw mushrooms on rice substrates had a diameter of approximately 46 cm. In this study, treatments were five baskets each as rice straw + mushroom spawn (T1), rice straw + Pb + mushroom spawn (T2) and rice stubble + Pb + mushroom spawn (T3). Mushroom cultivation methods in baskets followed the work of Ritnuch (2008). Each basket used approximately 1 kg of substrate divided into four parts. For the first layer, the substrate was spread in the baskets over some mushroom spawn. The process was repeated until four layers were established in each basket. Approximately 2 L of water was then added in all baskets. The five replicate baskets were covered by a chicken coop and enveloped with plastic sheets. The plastic sheets were opened for air ventilation about 1–2 h in the fourth day and covered again in the same day.

Substrate samples included rice straw and stubble with Pb accumulation and rice straw without Pb accumulation. All rice straw and stubble with Pb accumulation were collected from a paddy at Klity village, Thong Pha Phum District, Kanchanaburi Province. A paddy was divided into five sites for collecting substrates. Samples were collected in the center and at four angles of the paddy. In each site, 10 kg of rice straw and stubble were mixed and used in combination as a substrate sample. In controls, 10 kg of the rice straw without Pb were collected from a cattle farm at Kasetsart University, Kamphang Saen Campus, Nakhon Pathom Province. Straw mushroom samples were harvested from all baskets in the egg and mature stages. Mature stages were collected in two parts as pileus and stalk.

Substrate and straw mushroom were cut and washed thoroughly with running tap water. Straw mushrooms were trimmed using a stainless steel knife. All samples were dried at ambient temperature for 24 h, then dried again in a hot air oven at approximately 105°C for 24 h. Dried samples were homogenized in a blender, sifted using a sieve size of 1 mm and stored in pre-cleaned (washed thoroughly with deionized water and dried with a hot air oven at approximately 60°C for 24 h) polyethylene bags. Approximately 0.5 g of a substrate and straw mushroom samples were digested with 10 mL of HNO_3_ (65 %) and 5 mL of HClO_4_ (70 %) (or mixture at the ratio of 2:1) on a hot plate at 200°C for 1–2 h, filtered with Whatman grade no. 42 and finally diluted to 100 mL with deionized water. A blank digest was prepared in the same way. The Pb content of the acid extract was determined with flame atomic absorption spectrometry (FAAS) (Komárek et al. 2007; Panich-pat and Srinives 2009; Yamaç et al. 2007; Zhu et al. 2011).

Straw mushroom samples were fixed in 5 % glutaraldehyde in 0.1 M phosphate buffer and postfixed in 2 % osmium tetroxide. They were dehydrated though a graded series of ethanols (30 %, 50 %, 70 %, 80 %, 90 %, 95 %, and 100 %) and embedded in Spurr’s resin. Ultrathin sections were cut with a glass knife and stained in 10 % uranyl acetate and lead citrate, then examined under a transmission electron microscope (TEM) (Panich-pat and Srinives 2009).

Differences in Pb content among the straw mushroom samples were tested by analysis of variance (ANOVA) at significance level *p* ≤ 0.05. Analysis of Pearson’s correlation analysis was done to compare Pb accumulation in straw mushrooms and substrates.

## Results and Discussion

Table 1 provides Pb concentrations in the analyzed substrates before and after cultivation. All substrate samples were determined on a dry weight basis. The highest pre-cultivation Pb concentrations were recorded in T3 (253.71, 367.43 and 445.35 mg kg^−1^ in crop 1, crop 2 and crop 3, respectively). Lowest Pb concentrations were recorded on T1 (BD in all crops). Results showed Pb concentration of T3 > T2 > T1 indicating that Pb was accumulated in rice stubble more than straw (Panich-pat and Srinives 2009). In addition, Pb concentrations in substrate decreased after mushroom cultivation because mushrooms were able to accumulate metals from the substrate or environment (Kalač 2010; Nilanjana 2005).

**Table 1:** Mean lead accumulation in substrate (results represent means of five replicates ± SD, mg kg^−1^)

Treatment	Crop 1		Crop 2		Crop 3	
	Before cultivation	After cultivation	Before cultivation	After cultivation	Before cultivation	After cultivation
T1	BD	BD	BD	BD	BD	BD
T2	13.00 ± 1.76	11.69 ± 1.61	11.72 ± 2.51	7.15 ± 1.35	16.00 ± 2.90	11.59 ± 1.16
T3	253.71 ± 50.91	219.36 ± 4.63	367.43 ± 130.75	325.74 ± 77.50	445.35 ± 189.64	410.07 ± 189.70

BD = Below detection limits (0.001 mg kg^−1^)

Results of Pb concentration analyses of straw mushrooms appear in Table 2. Crops 1, 2 and 3 had egg stage and pileus Pb concentrations exceeding the EU acceptable standard of 3 mg kg^−1^. Therefore consumers should not consume these straw mushrooms. In addition, results suggest that mushrooms and other macrofungi can act as a biosorbent removing Pb from plant substrates or other components in an ecosystem (Kalač 2010; Nilanjana 2005).

**Table 2:** Mean lead concentrations in straw mushroom (results represent means of five replicates ± SD, mg kg^−1^)

Crop	Day of cultivation	Lead concentration								
		Egg stage			Pileus			Stalk		
		T1	T2	T3	T1	T2	T3	T1	T2	T3
1	10	BD	–	–	BD	–	–	BD	–	–
	13	BD	BD	0.396 ± 0.20x	BD	0.120 ± 0.21x	1.475 ± 0.81x	BD	BD	0.004 ± 0.01x
	16	BD	0.388 ± 0.86x	0.460 ± 0.30x	BD	0.997 ± 0.67y	3.346 ± 2.37x	BD	0.102 ± 0.23x	0.016 ± 0.04x
	19	BD	1.197 ± 0.24y	2.202 ± 1.02y	BD	2.511 ± 0.24z	3.696 ± 2.05y	BD	0.262 ± 0.29x	0.688 ± 0.21y
2	10	BD	–	–	BD	–	–	BD	–	–
	13	BD	0.056 ± 0.13x	0.208 ± 0.20x	BD	0.380 ± 0.41x	2.240 ± 0.89x	BD	BD	BD
	16	BD	0.248 ± 0.37x	1.147 ± 0.73y	BD	1.656 ± 2.44xy	3.731 ± 1.54xy	BD	BD	0.044 ± 0.10x
	19	BD	1.632 ± 0.34y	3.693 ± 0.52z	BD	2.686 ± 1.00y	5.072 ± 1.78y	BD	0.068 ± 0.15x	0.476 ± 0.29y
3	10	BD	–	–	BD	–	–	BD	–	–
	13	BD	0.018 ± 0.04x	0.200 ± 0.09x	BD	0.322 ± 0.25x	1.321 ± 0.59x	BD	BD	BD
	16	BD	0.300 ± 0.09x	0.676 ± 0.26x	BD	0.594 ± 0.11x	1.678 ± 0.51x	BD	0.086 ± 0.12x	0.104 ± 0.15x
	19	BD	1.568 ± 0.46y	1.607 ± 0.70y	BD	2.472 ± 0.90y	3.932 ± 1.86y	BD	0.130 ± 0.25x	0.354 ± 0.06y

BD = Below detection limits (0.001 mg kg^−1^)

x, y, z = Values in the same column followed by the same letter are not significantly differently according to DMRT (*p* ≤ 0.05)

The results of this research indicate that several factors influenced Pb accumulation in straw mushrooms. These included the age of the fruiting body, mycelium, and the gross morphology of straw mushroom. The age of the fruiting body generally correlated with Pb accumulation in straw mushrooms (Table 2; cultivations 10–19) because mature stages of straw mushroom absorbed and transported Pb from substrate to the fruiting body. Several other researchers reported higher metal concentration in younger fruiting bodies of mushrooms, which explained the transport of a metal from mycelium to fruiting body during fructification. Thus, an increase in the mass of the fruiting body was associated with the concentration of metals (Ita et al. 2006; Kalač 2010; Kalač and Svoboda 2000).

The age of mycelium was very important to Pb accumulation in straw mushrooms because the Pb concentration in straw mushrooms increased with increasing age of the mycelium (Table 2), since most of the Pb granules accumulated in the cell wall of mycelium. Some researchers indicated that the level of metal in fruiting bodies of mushroom was affected by the age of mycelium and interval fructification (Aloupi et al. 2012; Hammond 1979; Jain et al. 1989; Kalač and Svoboda 2000; Svoboda et al. 2000).

The morphology of mushrooms, particularly the pileus and stalk, also affected Pb accumulation. Results showed that Pb concentrated in the pileus more than in the stalk. This agreed with research of García et al. (2009) who studied Pb in edible mushrooms at Lugo province, Spain, and found Pb concentrations in the cap of mushrooms higher than among the rest of fruiting-body.

**Fig. 1:**
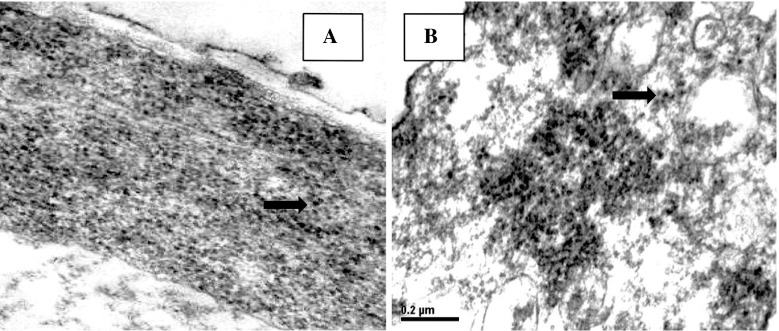
Lead granules (*arrow*) accumulated in the cell wall of straw mushroom **a** egg stage and **b** mature stage

The straw mushrooms in the egg and mature stages cultivated from T3, contained the highest Pb accumulation, were examined by TEM. Most of the Pb granules accumulated in the cell wall of mushrooms (Fig. 1). Cell wall consists of chitin, chitosan, glucan and amino polysaccharide. These components were associated with metal absorption (Gabriel et al. 2001; Mullen et al. 1992; Rome and Gadd 1991). Ariff et al. (1999) studied the kinetics and mechanism of Pb(II) biosorption by powderized *Rhizopus oligosporus*. They examined lead sorption, using TEM and found that during sorption most of the Pb was adsorbed on the surface of cell wall. Chitin and chitosan are important components of fungal cell walls, and both have been shown to sequester metal ions (Franco et al. 2004; Zhou et al. 2005). Pb uptake by *Rhizopus nigricans* was reported to be primarily because of the binding of Pb to the amine-N of chitin, which then acted as a nucleation site for the further deposition of Pb (Zhang et al. 1998). In addition, Bhanoori and Venkateswerlu (2000) studied cadmium (Cd) accumulation in *Neurospora crassa* and found Cd accumulation in *N*-acetylglucosamine polymer and chitin of the fungal cell wall.

In summary, Pb concentrations of straw mushroom cultivated in T3 exceeded the European Union standard for cultivated mushroom, hence, they might not be safe for human consumption. Therefore, consumers should be cautious about consuming mushrooms cultivated or grown in metal contaminated substrates.
